# When All Else Fails: A Rare Case of Postoperative Toxic Shock Syndrome Arising from Surgical Site Infection after Decompressive Neurectomy Successfully Treated with Angiotensin-2

**DOI:** 10.1155/2021/6698218

**Published:** 2021-02-10

**Authors:** Raghav Chandra, Samuel Gold, Casey Kohler, Jason Valladares, Sara A. Hennessy, Sneha G. Bhat

**Affiliations:** ^1^Department of Surgery, University of Texas, Southwestern Medical Center, 5323 Harry Hines Boulevard, Dallas, TX 75390, USA; ^2^Division of Burns, Trauma, Acute, and Critical Care Surgery, Department of Surgery, University of Texas, Southwestern Medical Center, 5323 Harry Hines Boulevard, Dallas, TX 75390, USA; ^3^Department of Anesthesia, University of Texas, Southwestern Medical Center, 5323 Harry Hines Boulevard, Dallas, TX 75390, USA

## Abstract

Toxic shock syndrome is a serious complication of *Streptococcus pyogenes* or *Staphylococcus aureus* infections associated with very high morbidity and mortality. Postoperative toxic shock syndrome is an extremely rare phenomenon which manifests as fevers, diffuse rash, septic shock, and death. We present the first reported case of toxic shock syndrome associated with a surgical site infection from a decompressive neurectomy for refractory migraines in a 41-year-old female as well as the first use of angiotensin-2 vasopressor therapy to treat persistent septic shock from toxic shock syndrome refractory to conventional therapies.

## 1. Introduction

Toxic shock syndrome (TSS) is a potentially lethal sequala of group A Strep (S*treptococcus pyogenes)* or *Staphylococcus aureus* infections which manifests with high fevers, diffuse erythematous rash, hypotension, and multisystem organ failure. Postoperative TSS is a rare clinical entity occurring in only 0.003% of surgical cases [1]. Treatment of TSS in these cases requires a high index of suspicion, early antibiotic administration, and aggressive hemodynamic support. In this report, we present the first reported case of TSS with septic shock, multisystem organ failure, and acute respiratory distress syndrome arising from a surgical site infection after decompressive occipital neurectomy for intractable migraine as well as the first report of successful use of angiotensin-2 vasopressor therapy to treat refractory septic shock from TSS.

## 2. Case Report

The patient is a 41-year-old female with a history of depression, obesity, tobacco abuse, previously treated cervical cancer, and longstanding migraines refractory to pharmacologic therapy who underwent a bilateral occipital zone release with neurectomy of the lesser occipital nerve and avulsion neurectomy of the third occipital nerve two weeks prior to her admission. A few days prior to her presentation, she complained of nausea, diarrhea, severe headaches, fever, neck stiffness, and burning and irritation at her posterior scalp incisions. She initially presented to a community hospital and was started on vancomycin and cefepime for presumed meningitis. She quickly developed respiratory insufficiency and hypotension requiring emergent intubation and vasopressor support. The patient was then transferred to our institution for a higher level of care.

She was admitted to the surgical intensive care unit in profound septic shock with acute renal and respiratory failure. Purulent discharge was expressed from the left cervical incision site, and she had a diffuse, macular, nonblanchable rash, and petechiae over the anterior neck, chest, abdomen, and lower extremities. Bloodwork demonstrated marked leukocytosis (white blood cell count: 25 × 10^9^ cells/L), acute renal failure (serum creatinine 3.08 mg/dL, with baseline of less than 1 mg/dL), and severe metabolic acidosis (pH: 7.09 and bicarbonate: 11 mEq/L) with significant base deficit (-19). Mean arterial pressures ranged from 40 to 60 mmHg prior to initiation of vasopressors. The incision was opened at bedside within two hours and debrided with evacuation of yellow-brown purulent discharge, but no evidence of nonviable tissue, fascial dehiscence, or dishwater fluid. Cultures from the surgical site, blood, and urine were obtained, and antibiotic therapy was broadened to vancomycin, cefepime, clindamycin, ampicillin, fluconazole, and acyclovir to cover for possible necrotizing soft tissue infection as well as meningitis.

Over the next several hours, the patient was persistently hypotensive despite being transiently responsive to aggressive fluid resuscitation with 17 liters of crystalloid and colloid infusions and vasopressor support with maximal doses of norepinephrine (0.3 mcg/kg/min), epinephrine (0.5 mcg/kg/min), vasopressin (0.04 units/min), and dopamine (2.5 mcg/kg/min) infusions. Stress-dose steroids were also administered. Continuous renal replacement therapy (CRRT) was initiated promptly to correct her profound acidosis (with improvement in pH to 7.35 after several hours) and to treat significant volume overload. Bedside transthoracic echocardiography demonstrated global hypokinesis, right ventricular dilation with mildly reduced function, and left ventricular ejection fraction of 45%. She developed thrombocytopenia (platelet count: 8 from 235 × 10^9^ cells/L) concerning for disseminated intravascular coagulation (DIC).

Given limited available options to further support her hemodynamics, we elected to use angiotensin-2 (Giapreza, La Jolla Pharmaceutical, San Diego, CA, USA), a novel vasopressor agent. Over the next 24 hours with angiotensin-2 therapy (maximum dose: 40 ng/kg/min) as well as CRRT, the patient was successfully weaned off dopamine, norepinephrine, and epinephrine. She developed progressive mottling and distal necrosis of her upper and lower distal extremities with progressively fainter arterial signals concerning for acute limb ischemia. This was likely due to her prolonged hypotensive state and significant vasopressor requirements including high doses of angiotensin-2. She had also developed acute respiratory distress syndrome (ARDS), secondary to septic shock, aggressive resuscitation, and pulmonary edema requiring high positive pressure ventilation, paralysis, epoprostenol, and prone positioning for several days while remaining on CRRT.

During this time, the patient's wound cultures grew *Streptococcus pyogenes* (group A *Strep*), and her constellation of symptoms (high fevers and fulminant multisystem organ failure including ARDS and acute renal failure, diffuse rash, and DIC) was consistent with streptococcal toxic shock syndrome. Antibiotics were narrowed to high-dose penicillin-G and clindamycin, and she received 3 days of intravenous immunoglobulin (IVIG) therapy.

Her pulmonary status improved after several days of proning therapy and continued CRRT. She was deemed stable enough to undergo cross-sectional imaging which demonstrated a left renal infarct, splenic infarct, and left deep femoral vein thrombosis. Multiple risk factors associated with these multifocal thromboses were present including obesity, tobacco abuse, multisystem organ failure, low-flow state, hypotension, multiple invasive lines, immobility, and high dose vasopressor therapy including angiotensin-2. No other obvious source of infection was identified. She was extubated on hospital day 11. Repeat echocardiography demonstrated improved left ventricular ejection fraction to 66% with residual mild anterior wall hypokinesis. Her distal extremities were found to be nonsalvageable, and she underwent bilateral mid-carpal amputations, left below-knee amputation, and right lower extremity Lis-Franc amputation. She underwent aggressive inpatient rehabilitation, has been discharged home, and is undergoing prosthetic evaluations.

## 3. Discussion

Toxic shock syndrome (TSS) is an infrequently encountered but highly lethal infectious syndrome in surgical patients. To our knowledge, this is the first report of TSS arising from a surgical site infection from a decompressive neurectomy.

The most common microorganisms associated with TSS are *Streptococcus pyogenes* (more frequently the M1 and M3 serotypes) and *Staphylococcus aureus*. TSS results from an overwhelming host response to bacterial superantigens. These are mitogenic exotoxins which bind to major histocompatibility complex class-II (MHC-2), CD-28 costimulatory receptor, and T-cell receptors resulting in constitutive activation of T-cells and proinflammatory cytokine release [2]. Fulminant release of TNF-*α*, IL-2, IL-6, and IFN-*y* promotes a severe septic response resulting in microvascular and macrovascular injury, DIC, and ultimately multisystem organ failure.

TSS presents with high fevers, tachycardia, hypotension, altered mental status, and a rapidly progressive, erythematous, violaceous rash with peripheral desquamation. There is usually an inciting primary infection, and multiple processes have been associated with TSS including infected foreign body, pneumonia, meningitis, scarlet fever, deep soft tissue infection, and necrotizing fasciitis [3]. Postoperative TSS is extremely rare, but has been documented in the literature as a complication from a broad range of procedures [4]. In a review of 390,000 cases performed between 1981 and 1993 at two hospitals, the incidence of TSS was 0.003% (12 cases) [1]. All of these cases were due to staphylococcal TSS [1]. In a separate series of 13 patients who developed postoperative staphylococcal TSS, a contaminated product (i.e., napkin and tampon) was only identified in 5 cases [4]. In this case, no such contaminated product was identified, further highlighting the highly unique presentation of postoperative streptococcal TSS.

Untreated TSS is uniformly fatal, and mortality rates up to 81% have been reported even with treatment [5]. A high index of suspicion with early initiation of antibiotics and fluid resuscitation is critical. Every effort must be made to identify and treat the inciting infectious source. In our case, this included debridement of the infected surgical site shortly after the patient's arrival to our institution. Broad-spectrum antibiotics covering MRSA and *Streptococcus* (i.e., vancomycin and linezolid) and gram-negative bacteria should be initiated until speciation occurs. Furthermore, clindamycin should be utilized to counteract systemic effects of toxin release. In this case, there was initial concern for meningitis, thus prompting empiric treatment for *Streptococcus pneumoniae*, *Neisseria meningitidis*, *Herpes*, and fungal species. Antibiotic coverage was narrowed to high-dose penicillin-G once *S. pyogenes* was isolated. Corticosteroid therapy for TSS is poorly studied, with isolated case series demonstrating a reduction in the duration of fever and illness severity [6, 7]. IVIG is often used as an adjunct for treatment of TSS. While its mechanism of action incompletely understood, IVIG is thought to neutralize superantigens and enhance opsonization and clearance of *S. pyogenes*. In a randomized double-blind trial of 21 patients with TSS treated with IVIG + standard therapy vs. standard therapy alone, those who received standard therapy alone had a 3.6-fold greater incidence of mortality at 28 days (although not statistically significant). However, there was a significant improvement in Sepsis-related organ failure assessment (SOFA) scores during hospitalization with use of IVIG [8]. We initiated IVIG therapy as soon as the presumptive diagnosis for TSS was established.

Hemodynamic instability and ultimate cardiovascular collapse are terminal manifestations of TSS and require aggressive fluid resuscitation and vasopressor support. Our patient suffered a fulminant course of TSS with multisystem organ failure and persistent hypotension refractory to maximum doses of norepinephrine, vasopressin, and dopamine. For this reason, we utilized a novel vasoactive agent, angiotensin-2. Angiotensin-2 acts on vascular smooth muscle cells to increase vasoconstriction, sympathetic tone, and production of aldosterone from the adrenal cortex, ultimately increasing mean arterial pressure. Angiotensin-2 was Food and Drug Association (FDA) approved in 2017 after the landmark ATHOS-3 trial demonstrated its efficacy in significantly improving mean arterial pressure while decreasing other vasopressor requirements in patients with refractory distributive shock [9]. The use of angiotensin-2 facilitated successful correction of our patient's refractory hypotension and stabilized her hemodynamic status. However, its use likely contributed to the development of acute arterial thromboses with renal and splenic infarcts and bilateral upper and lower extremity acute limb ischemia, ultimately requiring bilateral upper and lower extremity amputations. While this complication was likely multifactorial due to profound hypotension (untreated MAP as low as 40) requiring maximal vasopressor support, and extremely high inflammatory disease burden, the risk of vascular thrombotic events is significantly increased with angiotensin-2 compared to conventional therapy which may need further exploration [9, 10]. Indeed, in addition to increased incidence of infection and tachycardia, there was a higher incidence of thromboembolic events (13 vs. 5%) and peripheral ischemia (3.1 vs 1.9%) in patients treated with angiotensin-2 vs. placebo. Thus, the manufacturers recommend concurrent thromboembolic prophylaxis [9, 10]. Our patient is currently being evaluated and fitted for prostheses, but will be significantly limited in her mobility and daily activities. However, she has made a remarkable recovery after presenting to the hospital in moribund condition.

## 4. Conclusions

To our knowledge, this is the first case report of postoperative streptococcal toxic shock syndrome arising from a surgical site infection after an occipital neurectomy for refractory migraines. It is also the first reported case of postoperative TSS successfully treated using angiotensin-2 therapy for severe septic shock. Our case highlights that postoperative TSS can be fulminant with early and severe multisystem organ failure requiring a high level of care to successfully support and treat these patients. It also highlights the therapeutic potential of angiotensin-2 for refractory septic shock, while reflecting the severity of its potential thrombotic complications. In summary, toxic shock syndrome is a potentially devastating complication from severe staphylococcal or streptococcal infections. Postoperative toxic shock syndrome is extremely rare and requires a high index of suspicion for early diagnosis and treatment. Broad-spectrum antibiotics, source control, IVIG, and aggressive hemodynamic support with fluids and vasopressors are crucial for the treatment of postoperative toxic shock syndrome.

## Data Availability

The underlying data were abstracted from the electronic medical record at the University of Texas, Southwestern Medical Center.
